# Circulating tumor DNA: a revolutionary approach for early detection and personalized treatment of bladder cancer

**DOI:** 10.3389/fphar.2025.1551219

**Published:** 2025-03-21

**Authors:** Yan Zhou, Rongzhong Wang, Mingtang Zeng, Sijia Liu

**Affiliations:** ^1^ Department of Pharmacy, West China Hospital, Sichuan University, Chengdu, China; ^2^ West China Hospital, Sichuan University, Chengdu, China

**Keywords:** bladder cancer, circulating tumor DNA, early detection, personalized treatment, liquid biopsy

## Abstract

Bladder cancer is a malignant tumor with a high global incidence and recurrence rate. Traditional diagnostic methods, such as cystoscopy and urine cytology, have limitations in sensitivity and specificity, particularly in detecting low-grade bladder cancer. Circulating tumor DNA (ctDNA) offers a non-invasive alternative, reflecting tumor genetic characteristics through blood samples. It demonstrates high sensitivity and repeatability, making it a promising tool for early detection, recurrence monitoring, and treatment evaluation. Clinical studies have shown that ctDNA not only detects tumor burden but also captures dynamic tumor mutations, aiding in personalized treatment strategies. Despite its potential, clinical implementation of ctDNA faces challenges, including optimization of detection techniques, standardization, and the cost of testing. This paper explores the role of ctDNA in advancing bladder cancer diagnosis and treatment, with a focus on refining its clinical application and guiding future research toward improved patient outcomes.

## 1 Introduction

Bladder cancer is one of the most common malignancies of the urinary system worldwide, with high incidence and recurrence rates, presenting significant challenges to global health systems. Globally, there are approximately 570,000 new cases and 210,000 deaths from bladder cancer each year, with these numbers steadily increasing (Lopez-Beltran et al., 2024; van Hoogstraten et al., 2023). The incidence of bladder cancer exhibits notable regional disparities. Higher rates are observed in developed countries, which may be attributed to greater awareness, access to advanced diagnostic methods, and better treatment options. In contrast, lower-income regions face significant challenges in early detection and treatment, leading to worse outcomes (Teoh et al., 2020). Pathologically, about 70%–75% of bladder cancer patients are diagnosed with non-muscle invasive bladder cancer (NMIBC), and 20%–25% with muscle-invasive bladder cancer (MIBC) (Lopez-Beltran et al., 2024). While most NMIBC patients achieve favorable outcomes through transurethral resection of bladder tumor (TURBT), the disease’s high recurrence rate (21%–43%) and multifocal nature necessitate ongoing monitoring, early diagnosis, and personalized treatment to effectively manage the disease (Zhao et al., 2023).

Currently, the diagnosis and follow-up of bladder cancer primarily rely on cystoscopy and urine cytology (Sbizzera et al., 2022). These methods, while widely used, have limitations, including low sensitivity of urine cytology for detecting low-grade urothelial carcinoma and carcinoma *in situ*, often leading to delayed diagnoses (Bansal et al., 2021). For instance, urine cytology has a sensitivity of only around 50%–60% for low-grade tumors, which can significantly impair early detection (Yafi et al., 2015). Moreover, while biomarker tests like NMP22, BTA, and FISH have been employed for diagnosis, their sensitivity and specificity remain insufficient for clinical needs, with performance varying across patient populations and tumor stages (Zhao et al., 2023). These limitations underscore the pressing need for more sensitive, non-invasive diagnostic methods for improved early detection and monitoring of bladder cancer.

**FIGURE 1:**
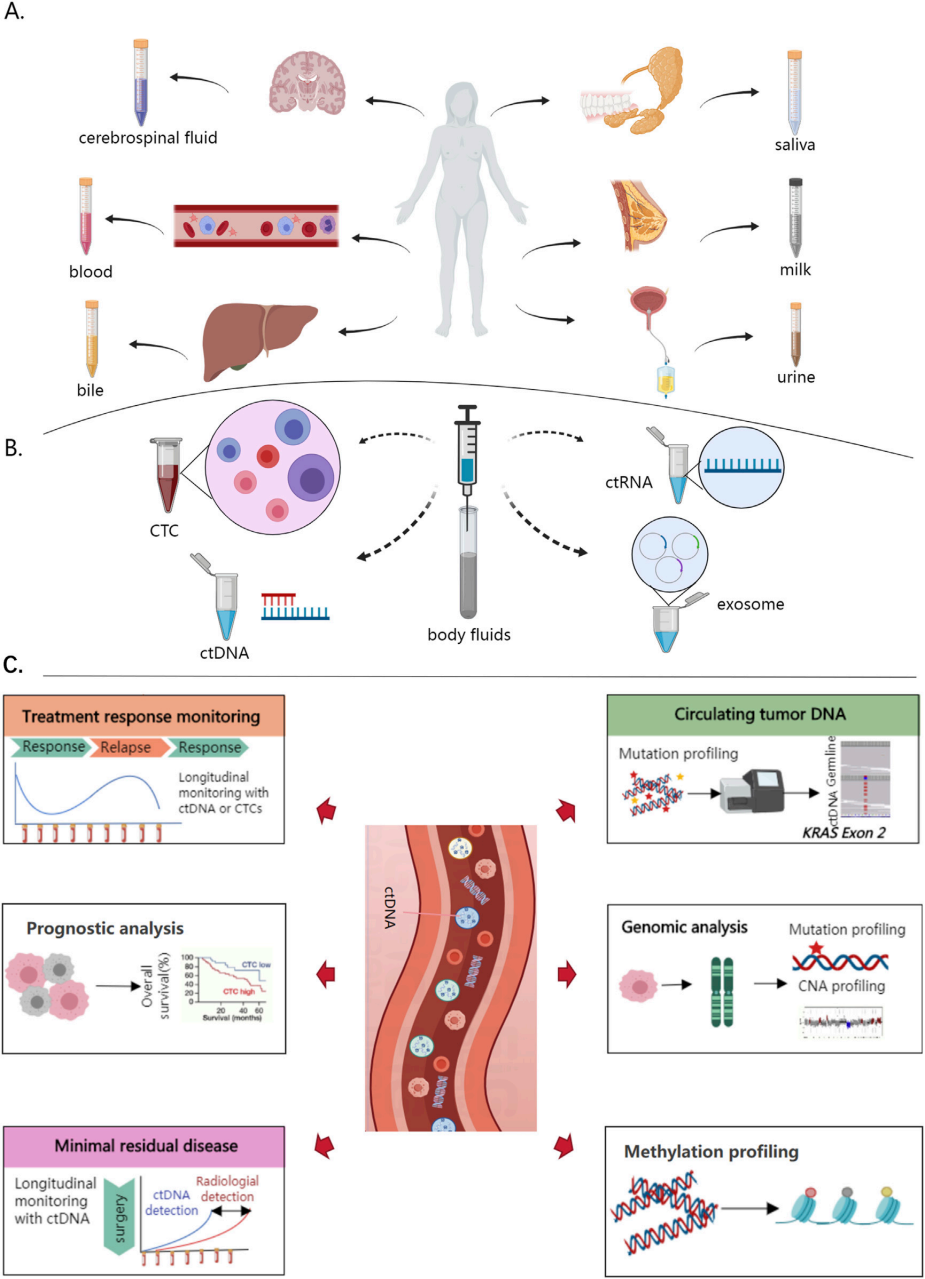
Liquid biopsy applications in cancer **(A)** Sampling of various bodily fluids for liquid biopsy, including cerebrospinal fluid, blood, bile, saliva, milk, and urine, each of which can provide critical biomarkers pertinent to different body systems. Cerebrospinal fluid reflects central nervous system health, blood is crucial for systemic analysis, bile indicates hepatic conditions, saliva offers a non-invasive diagnostic tool, milk provides insights into mammary gland states, and urine helps assess urinary tract health **(B)** Identification of advanced biomarkers through liquid biopsy, utilizing circulating tumor cells (CTCs), circulating tumor DNA (ctDNA), circulating tumor RNA (ctRNA), and exosomes, which are isolated from body fluids using sophisticated molecular techniques. These biomarkers, present in the bloodstream, enable non-invasive detection of tumor-specific genetic and epigenetic changes **(C)** Applications of ctDNA in bladder cancer. Treatment response monitoring by tracking ctDNA levels to evaluate therapy effectiveness and detect relapses, prognostic analysis to predict survival rates and disease progression with higher ctDNA levels indicating poorer prognosis, minimal residual disease detection post-surgery to identify residual cancer cells early, and mutation profiling for identifying specific genetic mutations like KRAS exon two for personalized treatment approaches. Genomic and methylation profiling of ctDNA further provide comprehensive genetic and epigenetic insights into tumor characteristics, aiding in precise and targeted therapeutic strategies.

In recent years, liquid biopsy methods, including circulating tumor cells (CTCs) and circulating tumor DNA (ctDNA), have gained increasing attention as tools for comprehensive cancer management (Figures 1A,B) (Markou et al., 2022). They hold significant promise for early diagnosis, treatment evaluation, and prognosis monitoring in bladder cancer, with ctDNA in particular showing great potential (Figure 1C) (Carrasco et al., 2023). ctDNA refers to tumor-specific DNA fragments released by cancer cells into the bloodstream, offering a non-invasive means of reflecting tumor burden, mutational status, and treatment response (Tivey et al., 2022). Compared to traditional tissue biopsy, ctDNA provides advantages such as high sensitivity, non-invasiveness, and repeatability, making it an excellent candidate for personalized diagnosis and treatment monitoring (Zhang et al., 2021). Although ctDNA has demonstrated superior sensitivity and specificity compared to traditional biomarkers such as NMP22 and FISH, there is potential for overlap and synergy between these methods. For example, ctDNA can provide a comprehensive genomic profile of the tumor, while NMP22 and FISH offer specific molecular and chromosomal insights that may complement ctDNA findings. Integrating ctDNA with traditional biomarkers could enhance diagnostic accuracy, particularly in cases where tumor heterogeneity or low tumor burden complicates detection (Garcia-Murillas et al., 2025; Breen et al., 2015). This article reviews the recent advances in ctDNA applications in bladder cancer, focusing on its potential and challenges in early diagnosis, treatment monitoring, and prognosis assessment, thereby offering a foundation for future research and clinical practice.

## 2 The biological basis of ctDNA

Cell-free DNA (cfDNA) is a biomarker released into the bloodstream by various cells, particularly through processes such as apoptosis or necrosis. These cfDNA fragments are typically small, around 166 base pairs in size, and are generally protected by nucleosomes (Newman et al., 2014; Snyder et al., 2016). Tumor cells also release cfDNA that carries tumor-specific genetic mutations, known as ctDNA (Leary et al., 2012). While the release of cfDNA from dead cells is well understood, recent research has provided evidence for the active release of cfDNA from living cells as well (Grabuschnig et al., 2020). This active release occurs through mechanisms such as exocytosis or secretion by viable tumor cells, and is thought to involve membrane vesicles like exosomes, microparticles, or apoptotic bodies. These mechanisms enable ctDNA to continuously circulate in the bloodstream, even in the absence of cell death, potentially reflecting real-time changes in tumor dynamics (Hu et al., 2021).

The release of ctDNA is not solely driven by cell death but is also influenced by the tumor microenvironment (TME), which encompasses various factors such as hypoxia, inflammation, and immune responses (Sánchez-Herrero et al., 2022). Apoptosis and necrosis, two major forms of cell death within the TME, are particularly important in ctDNA release. In apoptotic cells, nuclear fragmentation leads to the formation of nucleosome-protected cfDNA fragments, which are then released into the bloodstream. Necrotic cells, on the other hand, release larger DNA fragments, and this process may contribute to higher levels of cfDNA during tumor necrosis (Stejskal et al., 2023). The tumor microenvironment plays a critical role in modulating these processes, influencing both the quantity and quality of cfDNA released. For instance, inflammation within the TME can enhance the release of cfDNA from both viable and non-viable tumor cells, thereby increasing ctDNA levels.

CfDNA has a short half-life of typically less than 2 h, making it an ideal biomarker for real-time monitoring of tumor burden and treatment response (Diehl et al., 2008). This short half-life means cfDNA levels can rapidly fluctuate, providing timely insights into tumor status, particularly in patients with high tumor burden or aggressive cancers, where ctDNA detection is more frequent and pronounced (Diehl et al., 2008; Addanki et al., 2022). For instance, ctDNA levels were correlated with tumor burden in multiple cancer types, and increases in ctDNA levels were observed to correlate with disease progression or recurrence (Wan et al., 2017). However, it is important to note that the short half-life of cfDNA could pose limitations in slow-growing tumors, where the turnover of tumor cells may be less frequent, potentially leading to lower and less detectable ctDNA levels.

Nucleosomes, which protect cfDNA fragments, play a critical role in stabilizing ctDNA, making it detectable in the bloodstream. This protective mechanism has been applied diagnostically, particularly in assessing the presence of ctDNA mutations. For example, the nucleosome-protected DNA fragments have been utilized in liquid biopsy tests to identify specific mutations associated with cancer, such as mutations in the KRAS or TP53 genes, helping to guide personalized treatment strategies (Yin et al., 2021). Compared to other liquid biopsy biomarkers, such as CTCs or exosomal RNA, ctDNA offers several distinct advantages (Wang et al., 2024). CTCs, while providing direct evidence of viable tumor cells, are often more difficult to isolate and detect, particularly in patients with low tumor cell shedding. Exosomal RNA, on the other hand, may be less abundant and more variable across different tumor types (Jiang et al., 2019). ctDNA, due to its widespread presence and direct correlation with tumor genetics, offers a more reliable and sensitive means of monitoring tumor burden and therapeutic response. Furthermore, ctDNA can reflect not only the presence of genetic mutations but also changes in tumor dynamics, such as clonal evolution, which makes it a powerful tool for precision medicine (Wan et al., 2017).

## 3 ctDNA detection and diagnosis

The potential of ctDNA in urine for bladder cancer detection has been widely studied, particularly in the areas of TERT mutations and epigenetic modifications. Detection of TERT promoter mutations in urine has shown a sensitivity of 55.56% and a specificity of 100% (Jain et al., 2021). Using high-throughput sequencing technology for further analysis of common bladder cancer mutations, the sensitivity increased to 84%, with specificity ranging between 96% and 100% (Dudley et al., 2019). Additionally, the mutation frequencies of TERT and PLEKHS1 promoters were high at 75% and 46%, respectively (Dudley et al., 2019). High-throughput sequencing (HTS) plays a crucial role in detecting multiple mutations simultaneously, allowing for a more comprehensive analysis. The multiplexing capabilities of HTS enable the detection of various mutations in a single test, which enhances diagnostic efficiency and reduces the cost and time associated with traditional single-target methods. This allows for a broader understanding of the genetic landscape of bladder cancer, thereby improving detection accuracy and patient stratification (Li et al., 2023). In a study involving 355 bladder cancer patients and non-malignant hematuria patients, urine PCR detection of IQGAP3/BMP4 and IQGAP3/FAM107A showed a sensitivity of 71.0%, specificity of 88.6%, and an area under the curve (AUC) of 0.862 (Xu et al., 2019). These data further support the potential of urine ctDNA in bladder cancer detection. Another important research direction is the detection of methylation and copy number aberrations of urine ctDNA, which showed a sensitivity of 93.5% and a specificity of 95.8% for bladder cancer detection (Xu et al., 2019). Particularly, in a pathological analysis, the sensitivity for low-grade NMIBC detection was 84.2% (Xu et al., 2019). Furthermore, urine ctDNA has shown promising results in the detection of upper urinary tract tumors. By detecting methylation of specific bladder cancer-related methylation markers such as TERT promoter and ONECUT2 CpG sites, the method achieved a sensitivity of 94.0%, specificity of 93.1%, and an AUC of 0.961 in the validation dataset (Xu et al., 2020). Low-coverage whole-genome sequencing (WGS) technology for detecting urothelial cancers showed a sensitivity of 82.5%, specificity of 96.9%, and accuracy of 89.0% (Zeng et al., 2020). These results suggest that urine ctDNA detection has the potential to become a sensitive screening tool for bladder cancer.

While plasma ctDNA has been widely used for general cancer diagnostics, urine ctDNA offers several advantages specific to bladder cancer. Studies have shown that urine ctDNA detection for bladder cancer achieves comparable, if not superior, sensitivity and specificity. For example, urine ctDNA detection in bladder cancer has demonstrated high specificity (up to 100%) for TERT mutations, compared to plasma ctDNA, which has shown somewhat lower sensitivity in detecting bladder-specific mutation (Patel et al., 2017). Additionally, urine ctDNA offers a direct reflection of tumor burden in the bladder, which may improve the accuracy of detection, especially for lower urinary tract cancers (Christensen et al., 2017). However, plasma ctDNA is more widely applicable across various cancers, making it a versatile tool for liquid biopsy. In contrast, urine ctDNA is specifically valuable for diagnosing and monitoring bladder cancer.

The stable detection ability of ctDNA makes it of great value in guiding the emerging targeted therapy and immunotherapy for metastatic bladder cancer, especially in terms of targeted changes in the MAPK/ERK and PI3K/AKT/mTOR pathways (Vandekerkhove et al., 2017). In FGFR mutation studies, ctDNA is considered to play an important role in identifying FGFR variations and predicting response to FGFR inhibitors (such as rogaratinib, edafitinib, etc.) (Christensen et al., 2017; Guercio et al., 2023). Although clinical trials in patients with locally advanced or metastatic FGFR1/3 mRNA-positive urothelial carcinoma have not shown a significant advantage in response, *post hoc* analysis of patients carrying FGFR3 mutations has shown significantly higher objective response rates than the chemotherapy group, further validating the potential of ctDNA as a biomarker for treatment selection (Sternberg et al., 2023). Additionally, mutations in DNA damage repair (DDR) genes are also associated with the response of bladder cancer to platinum-based therapy (Plimack et al., 2015). Studies have shown that patients with DDR gene mutations have significantly increased response rates to platinum and other treatments, and the ERCC2 mutations detected in ctDNA are associated with increased sensitivity to platinum and improved progression-free survival (Vandekerkhove et al., 2021; Galsky et al., 2018). Overall, the genomic profile results of ctDNA not only provide theoretical basis for targeted therapy and immunotherapy of bladder cancer, but also solve the problem of insufficient primary tumor tissue samples.

In addition, ctDNA gene mutation detection, as a non-invasive method, can accurately monitor the gene mutation status in cancer patients’ body through blood samples, providing an important basis for the development of personalized treatment plans and prediction of treatment efficacy. Vandekerkhove et al. analyzed ctDNA samples from 104 patients with metastatic bladder cancer and found that the mutation characteristics of driver genes in ctDNA were highly consistent with those in tumor tissues, with a mutation consistency rate of 83.4% (Vandekerkhove et al., 2021). This consistency suggests that ctDNA could serve as a reliable surrogate for tumor biopsy, particularly in clinical settings where obtaining tumor tissue may be challenging or impossible. The high degree of mutation concordance between ctDNA and tumor tissues also facilitates treatment planning by ensuring that ctDNA mirrors the genomic features of the primary tumor, thus supporting decisions on targeted therapy (Vandekerkhove et al., 2021). Among them, seven cancer genes (ERBB2, ERBB3, PPARG, MYC, CCND1, CCNE1, MDM2) were detected to be amplified in both ctDNA and tumor tissues, indicating the reliability of ctDNA in reflecting the genomic features of tumors (Vandekerkhove et al., 2021). Furthermore, the detection of ctDNA is closely related to the prognosis of patients. Studies have shown that in patients receiving immunotherapy, FGFR3 mutation is associated with a shorter progression-free survival (PFS), suggesting that mutations in ctDNA may be an important indicator for prognosis evaluation.

The mutation profiles of FGFR3 and TERT in ctDNA not only provide insights into tumor heterogeneity but also reflect the clonal evolution of bladder cancer. Clonal evolution, driven by selective pressures such as therapy or immune surveillance, can lead to the emergence of resistant subclones. For instance, FGFR3 mutations, which are often early events in bladder carcinogenesis, may persist as dominant clones or evolve into subclones with additional genomic alterations under therapeutic pressure (Murtaza et al., 2013). Studies have shown that ctDNA can capture this dynamic process by detecting both dominant and minor subclonal mutations, offering a real-time snapshot of tumor evolution (Abbosh et al., 2017). For example, the presence of FGFR3 mutations in ctDNA has been associated with the emergence of resistance to FGFR inhibitors, often due to secondary mutations in the FGFR3 kinase domain or activation of alternative signaling pathways such as PI3K/AKT/mTOR (Nogova et al., 2017). Similarly, TERT promoter mutations, which are highly prevalent in bladder cancer, are often clonal and present in both primary and metastatic lesions, suggesting their role in early tumorigenesis and potential utility as stable biomarkers for monitoring disease progression (Rachakonda et al., 2013).

The high mutational rate of bladder cancer makes it an ideal candidate for ctDNA analysis, and the level of ctDNA can directly reflect the tumor burden (Todenhöfer et al., 2018; Christensen et al., 2023). Therefore, there is a need for highly sensitive screening methods to detect traces of ctDNA in the early stages and when the tumor burden is low. Studies have found that KRAS2 mutations in the plasma DNA of healthy individuals are closely related to the occurrence of bladder cancer, and this mutation can even be detected before clinical diagnosis (Gormally et al., 2006). The detection of TERT promoter mutations also shows the potential for early diagnosis. Research has shown that this mutation can be detected in urine samples up to 10 years before bladder cancer diagnosis, with a specificity of up to 100%, although the sensitivity is relatively low at 46% ± 7% (Hosen et al., 2020). In addition, the detection of urine tumor DNA (utDNA) is superior to traditional cystoscopy and cytology examinations, with a sensitivity of 91% and a specificity of 96% (Green et al., 2021). Despite the promising potential of ctDNA, there are limitations associated with its detection. The sensitivity of ctDNA detection in early-stage cancers remains a challenge, with detection rates in stage I cancers being as low as 16.8% in multi-cancer early detection tests (Klein et al., 2021). This low sensitivity may be attributed to the relatively small quantities of ctDNA released in the early stages of cancer, particularly in cases with low tumor burden. Moreover, false positives in non-malignant conditions, such as infections or inflammatory diseases, may complicate the interpretation of ctDNA results (Hill et al., 2021). To address these issues, there is a need for more sensitive detection technologies that can identify trace amounts of ctDNA in the early stages of cancer while minimizing false positives. In addition, improvements in the specificity of ctDNA markers are necessary to enhance diagnostic accuracy, especially when ctDNA is detected in non-cancerous conditions. In the future, ctDNA is expected to play a greater role in the early screening and diagnosis of bladder cancer as detection techniques and methods continue to be optimized.

## 4 The role of ctDNA in the prognosis of bladder cancer

CtDNA has shown significant value in predicting the prognosis of bladder cancer patients. In a cohort of 68 patients with MIBC who underwent radical cystectomy (RC) after neoadjuvant chemotherapy (NAC), a positive ctDNA status was highly associated with poorer recurrence-free survival (RFS) (HR = 4.2, 95% CI: 1.8–9.8, p < 0.001). Among patients tested for ctDNA before RC following NAC, 84% (52/62) were ctDNA negative, with 81% (42/52) achieving pathologic complete response (PCR), while patients with positive ctDNA did not reach PCR (Shen et al., 2021). Further analysis revealed that postoperative ctDNA positivity was not only closely associated with higher pathological staging (e.g., ≥pT3 disease, p = 0.003) but also predicted a high risk of bladder cancer recurrence (Carrasco et al., 2022). Compared to traditional imaging modalities (e.g., CT/MRI), which detect recurrence at a median of 6–12 months post-surgery, ctDNA positivity identified recurrence with a median lead time of 3 months, offering a critical window for early intervention (Christensen et al., 2019). The study found that most patients who tested positive for ctDNA after RC eventually experienced recurrence, and these patients were usually ctDNA positive at initial diagnosis.

The prognostic utility of ctDNA dynamics (e.g., clearance or persistence) surpasses conventional histopathology in predicting long-term outcomes. For instance, in patients with persistent ctDNA post-NAC, the risk of recurrence was 8.1-fold higher than in those with ctDNA clearance (95% CI: 3.1–21.3, p < 0.001), whereas pathological staging alone showed weaker discrimination (HR = 2.1, p = 0.08) (Christensen et al., 2019). Although some patients achieve ctDNA clearance during treatment, they may not show a complete pathological response to NAC, underscoring the discordance between molecular and histological assessments (Christensen et al., 2019). The clinical relevance of ctDNA is further supported by the IMvigor010 randomized Phase III trial. Among 214 patients (37%) who tested positive for ctDNA after RC, both disease-free survival (DFS) (HR = 2.5, 95% CI: 1.7–3.6, p < 0.001) and overall survival (OS) (HR = 3.0, 95% CI: 1.9–4.7, p = 0.002) were significantly worse compared to ctDNA-negative patients (Laukhtina et al., 2022). Notably, ctDNA-positive patients receiving atezolizumab adjuvant therapy showed improved survival (median OS: 25.6 months vs 15.8 months with observation; p = 0.02), whereas no benefit was observed in ctDNA-negative cohorts (p = 0.41), highlighting the role of ctDNA in stratifying therapeutic response (Laukhtina et al., 2022). Current evidence for the prognostic value of ctDNA is predominantly derived from MIBC cohorts. In NMIBC, ctDNA detection is challenged by lower tumor shedding and DNA concentrations, though preliminary studies suggest its potential in high-risk subtypes (e.g., T1 with carcinoma *in situ*) (Papadimitriou et al., 2023). Further validation is needed to clarify subtype-specific utility.

## 5 The role of ctDNA in detecting tiny residual lesions of bladder cancer

Minimal residual disease (MRD) refers to the state in which a small number of tumor cells still exist in the body of cancer patients after undergoing radical surgery, which is one of the main factors leading to recurrence and disease progression (Moding et al., 2021). MRD detection using liquid biopsy techniques, such as ctDNA, offers several advantages over traditional methods like imaging or tissue biopsy. While traditional methods rely on visualizing or sampling residual tumor tissue which may miss microscopic disease, liquid biopsy can detect minute levels of ctDNA shed by tumor cells into the bloodstream, providing a more sensitive and dynamic measure of MRD (Zhang et al., 2024). In recent years, ctDNA has been gradually applied in the detection and evaluation of MRD as an important biomarker. Jessica and colleagues developed a highly specific method for detecting the presence of ctDNA in early-stage NSCLC and bladder cancer patients from plasma without the need for tumor tissue but with comparable sensitivities to tissue-dependent approaches. The assay performance was tested using 163 pre-treatment clinical samples from patients with early-stage non small cell lung cancer (NSCLC) and bladder cancer and 133 self-declared healthy donors. Sensitivity for pre-treatment detection in NSCLC was 68.9% at 95% specificity (20/42 Stage I: 47.6%, 25/30 Stage II: 83.3%, 19/21 Stage III: 90.5%). Sensitivity for bladder cancer was 44.2% at 95% specificity (13/43 NMIBC: 30.2%, 18/27 MIBC: 66.7%). Additional development from larger cohorts and other tumor types is ongoing and data will be presented as available (Kurata et al., 2021). Survival analysis showed that ctDNA-positive patients had significantly shorter disease-free survival and overall survival than ctDNA-negative patients, further supporting the importance of ctDNA in MRD evaluation (Tivey et al., 2022).

The study by Christensen et al. further confirmed that preoperative ctDNA could clearly distinguish patients with or without residual disease, and patients who tested positive for ctDNA prior to neoadjuvant chemotherapy followed by cystectomy had a significantly higher risk of recurrence after surgery consolidation (Christensen et al., 2019). In ctDNA-positive patients, they observed an overall and 12-month recurrence rate of 75% (six of eight patients). In ctDNA-negative patients, the overall and 12-month recurrence rates were 11% (six of 55 patients; P < 0.001) and 7% (four of 55 patients; P < 0.001), respectively. The presence of ctDNA before cystectomy was associated with pathology at cystectomy as 100% of ctDNA-positive patients at this time point had residual tumor (stage ≥ T1) and/or lymph node metastases identified at cystectomy. Furthermore, 100% of patients (36 of 36) with pT0 at cystectomy were ctDNA negative (HR 12.0) (Christensen et al., 2019). In addition to plasma ctDNA, bladder cancer can transfer tumor DNA into urine, making urine ctDNA a potentially more suitable biomarker for MRD detection in bladder cancer due to its anatomical proximity to the tumor site. The direct shedding of tumor DNA into the urine may provide a more immediate and higher concentration of detectable ctDNA compared to plasma, which could enhance sensitivity and specificity in detecting residual disease (Mancini et al., 2020). Furthermore, results from urine cfDNA sequencing can be highly consistent with those obtained from tissue or plasma ctDNA sequencing, enabling the detection of MRD in urine through multi-omics sequencing analysis of urine cfDNA (Chauhan et al., 2023). However, it should be noted that while multi-omics sequencing for urine cfDNA shows promise, its validation across multiple clinical settings is still ongoing. More extensive studies involving diverse patient cohorts and standardized protocols are required to establish its robustness and reproducibility in clinical practice. These findings demonstrate the promising potential of ctDNA for the detection and evaluation of tumor MRD, providing important evidence for personalized treatment and promoting the precision and scientific nature of cancer therapies.

## 6 The role of ctDNA in recurrent monitoring of bladder cancer

CtDNA has shown significant value in the monitoring of bladder cancer recurrence. For example, Dudley et al. demonstrated that 91% of recurrent patients had detectable ctDNA in their urine, detecting recurrence 2.7 months earlier than clinical detection, providing an important opportunity for early intervention (Dudley et al., 2019). Christensen et al. further confirmed that ctDNA had a high sensitivity of 100% and specificity of 98% in monitoring metastasis and recurrence in locally advanced bladder cancer patients, with a median lead time of 96 days compared to imaging evaluations (Christensen et al., 2019). This lead time of 96 days offers a critical window for early therapeutic intervention, such as initiating adjuvant therapy or intensifying surveillance, which may improve outcomes by preventing disease progression or metastasis. For instance, in a study by Birkenkamp-Demtröder et al., early detection of ctDNA positivity prompted timely treatment adjustments, resulting in a 30% reduction in recurrence rates compared to standard clinical follow-up (p = 0.03) (Nordentoft et al., 2024). Carrasco’s study also supported this conclusion, finding that ctDNA could identify bladder cancer recurrence earlier. Among 12 recurrent or metastatic bladder cancer patients, MIBC patients had significantly higher levels of urine ctDNA compared to NMIBC patients, with changes detectable within 12–169 months before clinical diagnosis (Carrasco et al., 2022).

Urine ctDNA levels are not only closely related to bladder cancer progression, but can also assess tumor burden through the molecular tumor burden index (mTBI). A higher mTBI is positively correlated with tumor size, and is considered an independent prognostic factor for recurrence after tumor resection and immunotherapy (Zhang et al., 2021). The mTBI is calculated by quantifying the amount of ctDNA present relative to the total cfDNA in the sample. This ratio provides an estimate of the proportion of DNA that originates from the tumor, offering a molecular measure of tumor burden. Higher mTBI values indicate a greater tumor burden, which correlates with larger tumor size and more advanced disease. Validation of mTBI for widespread use is ongoing (Yi et al., 2021). Studies have demonstrated the prognostic value of mTBI, showing that it is a reliable indicator of tumor burden and a predictor of recurrence and progression (Yang et al., 2023). However, further multicenter prospective studies are required to fully validate mTBI across diverse patient populations and different stages of bladder cancer. Initial findings suggest mTBI can serve as an independent prognostic factor for recurrence after tumor resection and immunotherapy, but standardized methods for calculating and interpreting mTBI are needed for it to be adopted in clinical practice on a broader scale (Zhang et al., 2021).

CtDNA has also shown significant predictive value in NMIBC patients. Birkenkamp-Demtroder’s study showed that ctDNA levels in patients’ urine had already increased prior to a clinical recurrence or progression of the tumor. Further data showed that 48 of the 68 ctDNA-positive patients after resection experienced tumor progression or recurrence during follow-up (Birkenkamp-Demtröder et al., 2016). The ability to detect recurrence earlier through ctDNA monitoring may translate into improved survival outcomes, as early intervention during the lead time window could prevent the development of advanced disease, which is associated with poorer prognosis. In contrast, in MIBC, ctDNA monitoring is often used to track systemic disease and detect metastasis (Tan et al., 2018). MIBC patients typically have a higher tumor burden and a greater likelihood of systemic dissemination. Therefore, ctDNA levels in MIBC patients are often higher and can be used to monitor treatment response and detect metastasis earlier than traditional imaging methods. Studies demonstrated that ctDNA monitoring in MIBC patients had a high sensitivity and specificity, with a median lead time of 30–90 days compared to imaging evaluations (Carrasco et al., 2023; Carrasco et al., 2022). This longer lead time for early detection in MIBC is crucial for initiating systemic therapies that can control metastatic disease and potentially improve survival outcomes. While providing compelling evidence, the specificity and sensitivity of ctDNA in monitoring bladder cancer recurrence needs to be further validated through prospective multicenter randomized controlled trials. Despite the relatively small sample size and short follow-up time in the current studies, it has been tentatively demonstrated that urine ctDNA monitoring can improve the convenience of disease detection and improve patient outcomes through early identification.

## 7 ctDNA in the immune therapy of bladder cancer

CtDNA plays an important role in the immunotherapy of bladder cancer, especially in assessing the response of advanced cancer patients to adjuvant chemotherapy and immunotherapy. Recent studies have shown that the dynamic changes of ctDNA during treatment are closely related to therapeutic efficacy (Cheng et al., 2021). Christensen et al. demonstrated that the clearance of ctDNA during chemotherapy indicates a response to chemotherapy, providing a basis for additional chemotherapy cycles before radical cystectomy (Christensen et al., 2019). The clearance of ctDNA likely reflects the elimination of tumor-derived DNA from circulation, which is associated with a reduction in tumor burden and/or the effective immune-mediated elimination of cancer cells. These dynamic changes may signal the activation of immune responses or the cytotoxic effects of chemotherapy, suggesting that ctDNA clearance could serve as a surrogate marker for tumor regression and immune response in bladder cancer patients (Christensen et al., 2019). Furthermore, in the randomized Phase III trial IMvigor010, patients who were ctDNA-positive showed a significant improvement in overall survival when receiving adjuvant immunotherapy (HR = 0.59 (95% CI: 0.41–0.86)), while no significant change was observed in patients without detectable ctDNA. Patients with ctDNA clearance had more favorable survival outcomes during adjuvant immunotherapy (OS HR = 0.14 (95% CI: 0.03–0.59)), indicating that ctDNA clearance may be an important indicator for predicting the response of bladder cancer patients to immunotherapy (Powles et al., 2021).

The correlation between ctDNA clearance and immune response could be explained by the interaction between immune cells, such as T cells, and tumor antigens. When ctDNA is cleared, this suggests that the immune system has successfully recognized and attacked the tumor, leading to a reduction in circulating tumor DNA. Alternatively, ctDNA clearance may indicate the effectiveness of immune checkpoint inhibitors in boosting the anti-tumor immune response, facilitating tumor shrinkage and a better overall survival outcome (Zhang et al., 2020). Studies also suggest that MIBC patients who are ctDNA-positive at baseline may benefit from atezolizumab therapy (Szabados et al., 2022), with the ongoing IMvigor011 clinical trial evaluating the efficacy of atezolizumab in high-risk MIBC patients who are ctDNA-positive (Jackson-Spence et al., 2023). In the future, ctDNA is expected to become a predictive molecular biomarker for immunotherapy of urothelial carcinoma. In advanced urothelial carcinoma, monitoring the dynamic changes in ctDNA concentration can identify more treatment targets with higher sensitivity than single measurements. Research by Bratman et al. also confirmed a strong correlation between changes in ctDNA concentration and patients’ progression-free survival, overall survival, clinical response, among other outcomes (Bratman et al., 2020).

In addition, bladder cancer, as a cancer with a high tumor mutation burden (TMB), is closely related to immune therapy response (Klempner et al., 2020; Alexandrov et al., 2013; Maia et al., 2020). In a study of patients with locally advanced and metastatic urothelial carcinoma, responders had significantly higher median mutation burden than non-responders (Rosenberg et al., 2016). The study also found that in muscle-invasive bladder cancer, PD-L1, TMB, and DDR genes showed significant differences between patients who fully responded to pembrolizumab and non-responders, suggesting that TMB and DDR may be important predictors of treatment response (Necchi et al., 2018). The biological mechanism behind this finding could involve the enhanced neoantigen load in patients with high TMB, which increases the immune system’s ability to recognize and attack tumor cells. Moreover, defects in DDR pathways may lead to increased genetic instability, thereby enhancing the tumor’s immunogenicity and making it more susceptible to immune checkpoint inhibitors (Samstein et al., 2019). Assessment of protein and transcriptomic biomarkers, including PD-L1, TMB and the basal–squamous molecular subtype, provided insights into the biology associated with ctDNA positivity and response to atezolizumab, highlighting the relevance of immune and stromal contexture. The relationship between tumour-based biomarkers and ctDNA underscores that predictive biomarkers of response should be interpreted in the context of MRD (Powles et al., 2021). The anti-PD-1 therapy toripalimab also showed a good objective response rate and progression-free survival in patients with metastatic urothelial carcinoma with high TMB and PD-L1 expression (Sheng et al., 2022). Additionally, early ctDNA response to toripalimab treatment was associated with longer survival, while ctDNA copy number abnormality and cancer cell fraction could accurately predict patients’ response to immune therapy (Szabados et al., 2022; Zang et al., 2023). Moreover, the mean variant allele frequency (VAF) was shown to predict the efficacy of immune therapy. Studies found that dynamic changes in VAF were associated with tumor volume reduction, longer treatment duration, and were positively correlated with progression-free survival and overall survival (Raja et al., 2018; van Dorp et al., 2023). These findings further support the application prospects of ctDNA and VAF in immune therapy, indicating that they can not only help identify responders to immune therapy, but also provide important references for personalized treatment plans.

## 8 Clinical trials of ctDNA

The analysis of ctDNA has shown promising clinical utility in the management of patients with MIBC, particularly in improving patient outcomes, enhancing quality of life, and reducing treatment costs. The study by Christensen et al. proposed various clinical trial designs, with preliminary results suggesting that the absence of detectable ctDNA at the time of MIBC diagnosis is associated with better prognosis, potentially allowing these patients to avoid neoadjuvant chemotherapy. Conversely, patients with detectable ctDNA are at higher risk and should receive NAC, with ctDNA levels monitored throughout treatment (Christensen et al., 2019). A clearance of ctDNA would indicate chemosensitivity and might warrant an extension of treatment cycles. In contrast, persistent ctDNA detection could necessitate adjustments in the treatment plan, including considering early radical cystectomy in the absence of other systemic treatment options. Post-RC ctDNA monitoring has been shown to identify metastatic disease and could serve as a critical marker for initiating treatment, even when imaging does not reveal metastasis. In the context of metastatic disease management, ctDNA tracking provides real-time insights into therapeutic response, guiding modifications to treatment regimens (Vandekerkhove et al., 2021). These hypotheses require validation through prospective clinical trials before being incorporated into routine clinical practice.

Ongoing trials such as the TOMBOLA trial (NCT04138628), a single-arm, non-randomized phase II study expected to enroll 282 patients, are exploring the potential for early treatment initiation based on ctDNA detection. This trial aims to identify 127 ctDNA-positive patients and initiate atezolizumab therapy, with the primary endpoint being the assessment of response based on ctDNA status and CT scan results. Another study, the Imvigor011 trial (NCT04660344), builds on findings from the Imvigor010 trial and is a randomized, placebo-controlled phase III trial evaluating the efficacy and safety of atezolizumab in ctDNA-positive patients following RC, with a target enrollment of 495 patients and a primary endpoint of disease-free survival (Powles et al., 2021). Both trials investigate the potential survival benefits of early treatment based on ctDNA detection rather than traditional imaging criteria, with prospective evaluations being crucial for establishing the clinical utility of ctDNA in routine practice. Additionally, efforts are underway to develop an umbrella trial aimed at initiating targeted therapy based on ctDNA characterization in patients with locally advanced or metastatic bladder cancer, akin to current trials in metastatic castration-resistant prostate cancer (NCT03385655).

## 9 Conclusion and prospect

CtDNA has demonstrated significant potential in the diagnosis and management of bladder cancer, particularly in patients with MIBC. Current studies suggest that ctDNA can serve as an effective prognostic biomarker for early diagnosis, during neoadjuvant chemotherapy, and prior to radical cystectomy, with the ability to predict postoperative recurrence risk up to 3 months in advance. Furthermore, ctDNA holds promising potential in adjuvant therapy, particularly in predicting response to immune checkpoint inhibitors, although this application requires further validation through prospective studies. The potential of urinary ctDNA in monitoring recurrence of NMIBC also warrants attention. Compared to traditional pathological biopsies, ctDNA testing offers the advantages of being non-invasive, dynamic, and highly sensitive, facilitating personalized precision treatment. However, it is crucial to critically address the limitation of low sensitivity in ctDNA detection for early-stage bladder cancer. While ctDNA shows promise in detecting advanced bladder cancer, its sensitivity for detecting early-stage bladder cancer (especially in non-muscle-invasive bladder cancer, or NMIBC) remains a significant challenge. To address the issue of low sensitivity in early-stage bladder cancer detection, several strategies are being explored. First, advancements in HTS technologies, such as error-corrected sequencing and digital droplet PCR (ddPCR), have shown promise in detecting ultra-low levels of ctDNA with higher precision. These methods reduce background noise and improve the detection of rare mutations, thereby enhancing sensitivity. Second, the integration of multi-analyte approaches, combining ctDNA with other biomarkers such as protein markers or epigenetic modifications (e.g., DNA methylation), may improve early detection rates. In addition, the development of personalized ctDNA assays targeting patient-specific mutations or tumor-derived variants could further enhance sensitivity by focusing on known genomic alterations. These approaches, combined with machine learning algorithms to analyze complex genomic data, hold promise for improving the early detection of bladder cancer.

Similarly, there are challenges that need to be addressed for the clinical integration of ctDNA. These include issues related to testing costs, standardization of sampling techniques, and the regulation of testing procedures. Specifically, the lack of standardization in ctDNA detection protocols across different laboratories can lead to variations in results, hindering its clinical reliability. Inter-laboratory variability in the methods used for ctDNA extraction, quantification, and analysis remains a significant concern, which could affect the reproducibility of test outcomes. Furthermore, clinical adoption of ctDNA testing is often delayed by regulatory hurdles and financial constraints. These challenges need to be addressed through large-scale clinical trials and efforts aimed at standardizing detection protocols and reducing testing costs to fully realize the potential of ctDNA in improving patient outcomes. Overall, ctDNA is expected to become an integral component of bladder cancer diagnosis and treatment.
